# Supplementary material for: Suppression of Platelet-Derived Growth Factor Receptor-Alpha Overcomes Resistance to Trastuzumab through STAT3-Dependent IL-6 Reduction in HER2-Positive Breast Cancer Cells
Source: Biomedicines. 2023 Feb 23;11(3):675. doi: 10.3390/biomedicines11030675 (PMC10045855; doi:10.3390/biomedicines11030675)
Supplement: Supplementary file 1 [file biomedicines-11-00675-s001.zip › biomedicines-2133892-supplementary.pdf]

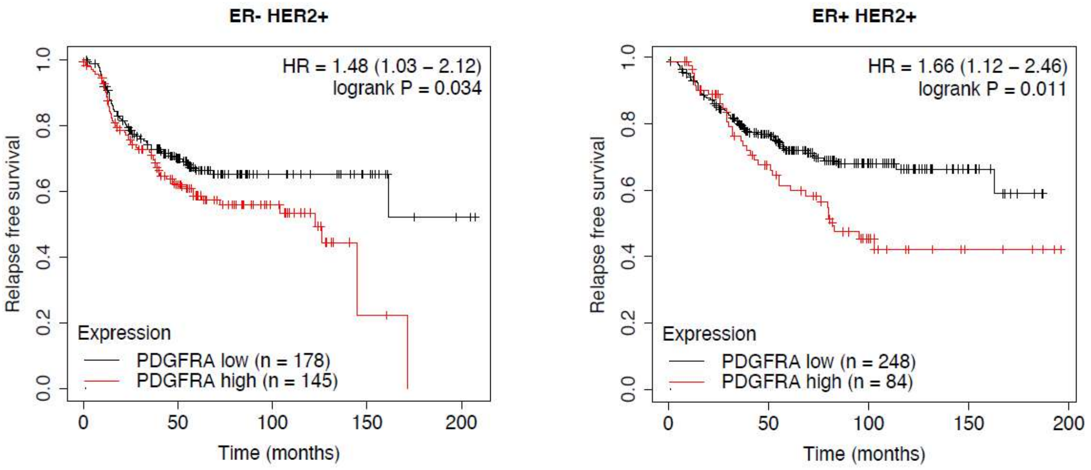

Supplementary Figure S1. Survival rates of ER- HER2+ and ER+ HER2+ breast cancer patients according to PDGFRA mRNA expression. Using the Kaplan-Meier plotter database, the alteration in relapse-free survival was analyzed according to PDGFRA mRNA expression in ER- HER2+ (low PDGFRA expression, n = 178; high PDGFRA expression, n = 145) and ER+ HER2+ (low PDGFRA expression, n = 248; high PDGFRA expression, n = 84) breast cancer patients. The patients were split by using the Auto select best cutoff option. Hazard ratio (HR) and 95% confidence intervals as well as log rank P were calculated and displayed on the webpage (https://kmplot.com/analysis/index.php?p=service&cancer=breast). P-value < 0.05 was considered to be statistically significant.

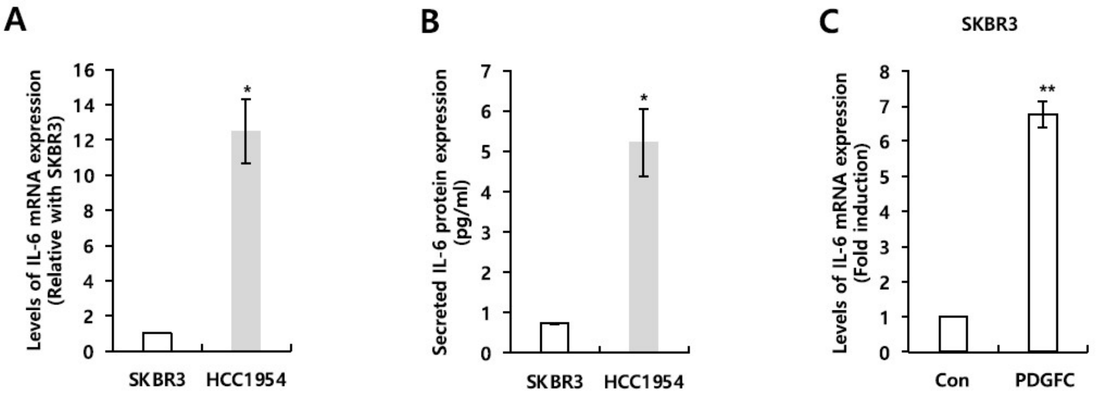

Supplementary Figure S2. IL-6 expression is upregulated in trastuzumab (TRZ)-resistant cells and PDGFC upregulates IL-6 expression. (A) IL-6 mRNA expression levels were

analyzed by qPCR. (B) Secreted IL-6 protein levels were analyzed by ELISA. (C) After serum starvation for 24 h, cells were treated with or without 25 ng/ml PDGFC for 48 h. IL-6 mRNA expression levels were analyzed by qPCR. Each experiment was carried out three independent times. Results are presented as mean ± SEM (*P < 0.05, **P < 0.01).

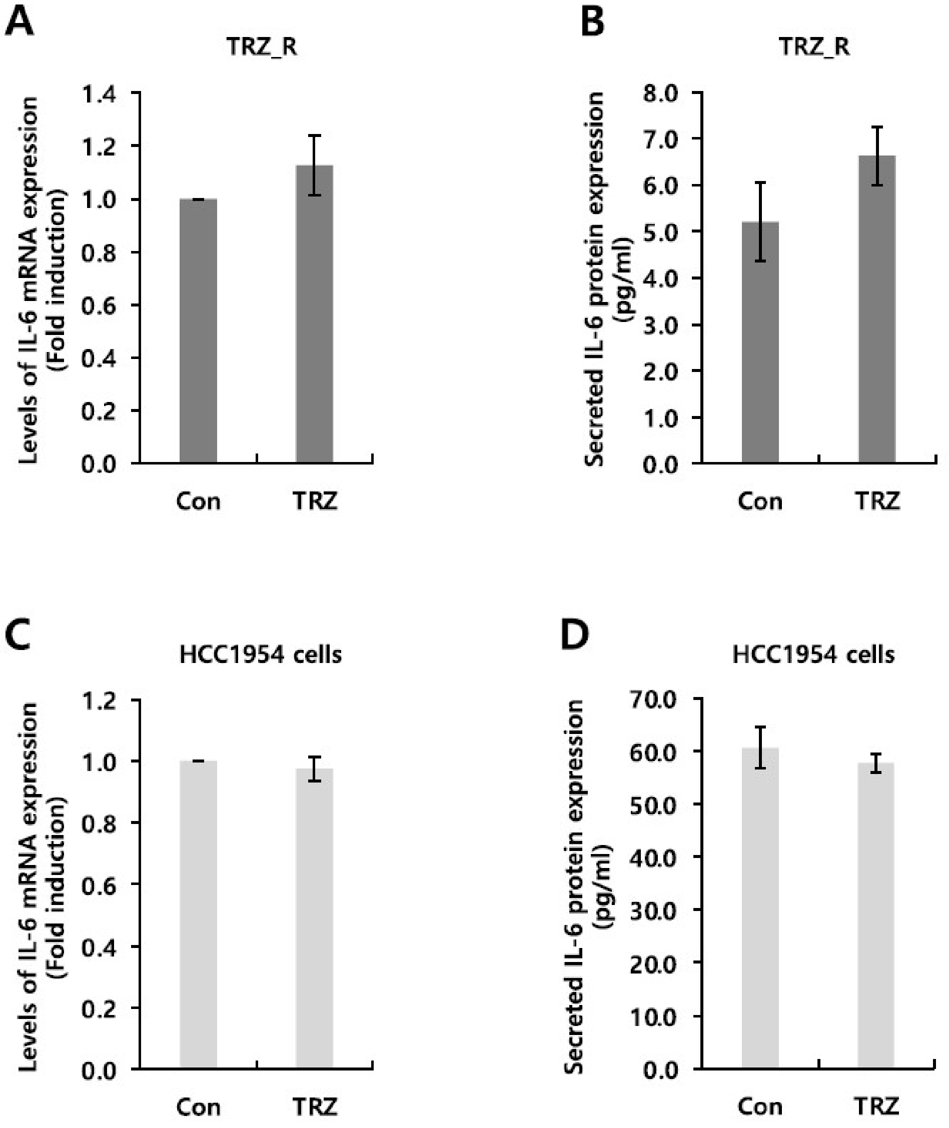

Supplementary Figure S3. TRZ does not affect IL-6 expression in TRZ-resistant breast cancer cells. After serum starvation for 24 h, cells were treated with or without 50 µg/ml TRZ for 48 h. (A, C) IL-6 mRNA expression levels were analyzed by qPCR. (B, D) Secreted IL-6 protein levels were analyzed by ELISA. Each experiment was carried out three independent times. Data are presented as mean ± SEM.
